# Water Quality in South Asia

**Published:** 2008-06

**Authors:** Stephen Luby

**Affiliations:** Programme on Infectious Diseases and Vaccine Sciences, ICDDR, B, GPO Box 128, Dhaka 1000, Bangladesh, Email: sluby@icddrb.org

Water, an essential compound for human life, all too often carries pathogens that cause disease. The article by Tambe *et al*. illustrates a community where half the time the available drinking-water was contaminated with organisms whose ecological niche is the human intestine (1). This finding is common throughout South Asia where both urban and rural water supplies are frequently contaminated with human faecal organisms. Although 85% of drinking-water in South Asia meets the target of the Millennium Development Goal of coming from an improved source (2), this water is, in fact, frequently contaminated with human faecal organisms (1,3,4). Indeed, the frequency of water contamination with human faeces is so common throughout South Asia that it is accepted as the norm. Those who can afford it buy bottled water (of dubious quality), and the majority are left to drink the available contaminated water. The commonality of this contamination risks preventing us from appreciating the seriousness of the problem. In the United States, at the beginning of the twentieth century, most large cities were served by municipal water suppliers that distributed untreated water throughout the city. Immediately following the introduction of effective water treatment, overall child mortality dropped by 46% in major US cities (5). Moreover, the microbiological contamination of water not only causes childhood death, but also repeated bouts of diarrhoea among children aged less than two years impair cognitive development and school performance among survivors (6,7). Thus, the failure to deliver clean water to the population of South Asia means more childhood deaths, less cognitive development, less educational achievement, and less economic growth.

Improving the water quality throughout South Asia is difficult. Interventions to improve the water quality are generally implemented by water engineers. The health impact of these interventions is less-commonly assessed. As an example, shallow tubewells were introduced and heavily promoted throughout Bangladesh in the 1970s as a way to improve the water quality of communities by shifting from heavily-contaminated surface water to microbiologically-cleaner groundwater. However, careful studies performed at the time when tubewells were introduced into Bangladesh concluded that there was no reduction in diarrhoea in households that used the new tubewells (8-10). This is not an isolated historical example. In a meta-analysis of studies of the community-based approaches to improve water supply, the type of intervention that Tambe *et al*. describe in their article, these interventions have not been associated with a significant reduction in diarrhoeal disease (11). We need to confront the dogmatism that current interventions represent improvement. We need more of what Tambe *et al*. have done, i.e. we need to evaluate the outcomes of interventions on water quality. We need to identify which interventions on water quality improve health and how these can be implemented at a large scale.

Importantly, the problem of water quality does not end with microbiological contamination. Groundwater, especially shallow groundwater, in many sites in South Asia is contaminated with dangerously-high levels of arsenic (12). Long-term exposure to the high levels of arsenic in drinking-water reduce child survival (13), and lead to cognitive impairment (14), cardiovascular diseases (15), and cancer (16). Although many approaches can remove arsenic from drinking-water, there is much less evidence available that these interventions can be introduced at scale and that their introduction is associated with a reduction in arsenic exposure of humans and improved health. The counter-intuitive findings of the minimal health impact on interventions designed to reduce microbiological contamination illustrate the importance of continued evaluation of the health impact of interventions to improve water quality.

In addition to human enteric pathogens and arsenic, drinking-water in South Asia can also be contaminated with industrial pollutants. The South Asian economies are developing. This means that a progressively smaller percentage of the workforce is engaged in agriculture, and more of the economy is devoted to industrial production. A common by-product of industrial outputs is industrial waste, a mélange of chemicals that pose substantial risk to human health. This is a particular risk to South Asia because the explosive industrial growth is occurring in the setting of weak rule of law. Companies that generate waste have a strong financial incentive to pollute. Paying extra to reduce contamination would lessen their profits. Part of the difficulty in understanding the scale of the problem of industrial pollution is that there are so many different chemicals involved that there is not a simple assay to assess industrial pollution. Public-health professionals can assist governments by improving surveillance for industrial contamination and making the reports of such contamination a routine part of reporting water quality for the country. In addition, efforts to reduce industrial pollution of water should be evaluated, and successful models promoted.

The water in South Asia is contaminated. Both increasing population and a warming climate risk further worsening the already-compromised situation. We need a renewed commitment to water quality. We in the research community can assist by conducting water-quality evaluations and by rigorous assessment of efforts to improve the water quality.
